# Inhibition of purinergic P2X receptor 7 (P2X7R) decreases granulocyte-macrophage colony-stimulating factor (GM-CSF) expression in U251 glioblastoma cells

**DOI:** 10.1038/s41598-020-71887-x

**Published:** 2020-09-09

**Authors:** Matthew Drill, Kim L. Powell, Liyen Katrina Kan, Nigel C. Jones, Terence J. O’Brien, John A. Hamilton, Mastura Monif

**Affiliations:** 1 Department of Neurosciences, Faculty of Medicine, Nursing and Health Sciences, Central Clinical School, Monash University, Melbourne, VIC Australia; 2 Department of Physiology, Melbourne University, Parkville, VIC Australia; 3 Department of Neurology, Alfred Health, Melbourne, VIC Australia; 4 Department of Neurology, Melbourne Health, Parkville, VIC Australia; 5 Department of Medicine, Melbourne University, Parkville, VIC Australia

**Keywords:** Cancer, Cell biology, Neuroscience, Oncology

## Abstract

Glioblastoma is the most aggressive form of primary brain cancer, with a median survival of 12–15 months. The P2X receptor 7 (P2X7R) is upregulated in glioblastoma and is associated with increased tumor cell proliferation. The cytokine granulocyte-macrophage colony-stimulating factor (GM-CSF) is also upregulated in glioblastoma and has been shown to have both pro- and anti-tumor functions. This study investigates the potential mechanism linking P2X7R and GM-CSF in the U251 glioblastoma cell line and the therapeutic potential of P2X7R antagonism in this setting. P2X7R protein and mRNA was demonstrated to be expressed in the U251 cell line as assessed by immunocytochemistry and qPCR. Its channel function was intact as demonstrated by live cell confocal imaging using a calcium indicator Fluo-4 AM. Inhibition of P2X7R using antagonist AZ10606120, decreased both GM-CSF mRNA (P < 0.05) and protein (P < 0.01) measured by qPCR and ELISA respectively. Neutralization of GM-CSF with an anti-GM-CSF antibody did not alter U251 cell proliferation, however, P2X7R antagonism with AZ10606120 significantly reduced U251 glioblastoma cell numbers (P < 0.01). This study describes a novel link between P2X7R activity and GM-CSF expression in a human glioblastoma cell line and highlights the potential therapeutic benefit of P2X7R inhibition with AZ10606120 in glioblastoma.

## Introduction

Gliomas are the most common primary malignancies of the central nervous system (CNS), representing 81% of malignant brain tumors in adults^1,2^. They are derived from glial cells, with an overall incidence between 3 and 6 per 100,000 persons^3–5^. They are classified into grades I–IV based on the criteria of histology and molecular features, as outlined by the World Health Organization^6^. Grade IV glioma, also known as glioblastoma, is the most common and most aggressive form, representing ~ 55% of all gliomas with an incidence rate between 0.59 and 3.69 per 100,000 persons^1,3,7^.

Glioblastoma commonly affects younger individuals, with the median age of presentation between 30 and 40 years^1^. The current standard therapy for glioblastoma includes surgical resection of the tumor, followed by radiotherapy and adjuvant chemotherapy using temozolomide (TMZ)^5,8^. Despite these treatments, glioblastoma is associated with high mortality: the average survival time is 14.6 months, and 5 year survival is < 5%^3,9^. Hence, targeted therapies that can specifically inhibit the growth of the glioblastoma cells and improve prognostic outcomes are urgently needed.

Neuroinflammation plays a key role in the pathogenesis of glioblastoma. It involves the activation and recruitment of immune cells such as microglia, monocytes and lymphocytes that can release a variety of bioactive factors that influence the tumor microenvironment. In glioblastoma, neuroinflammation is integral to the ongoing proliferation of the tumor^10,11^, with recruitment of large numbers of microglia and macrophages to the tumor site. These responses increase the release of bioactive factors that promote tissue invasion and tumor cell proliferation^12,13^, which can result in infiltration of glioblastoma cells into surrounding healthy tissue^11^. Therefore neuroinflammatory mediators are of key interest in understanding the link between neuroinflammation and glioblastoma propagation^14^.

The P2X purinoceptor 7 (P2X7R) is a key component in the neuroinflammatory cascade. P2X7R is a purinergic signaling receptor which binds adenosine triphosphate (ATP) to form a transmembrane cation channel^15^. It is expressed on a number of immune cells, such as monocytes, macrophages and microglia. P2X7R is upregulated in a number of human cancers, including both glioma cells and microglia in glioblastoma^16^ and its inhibition decreases tumor growth^17–19^. It has been shown that P2X7R activation interacts with multiple inflammatory intracellular signaling cascades including the NLRP3 inflammasome, the PI3K/Akt pathway, NFκB activation and subsequent release of Interleukin 1 beta (IL-1β)^15,20–25^. The P2X4 receptor has also been shown to potentially interact with the P2X7 receptor to activate these inflammatory pathways^26^. Recent studies have highlighted the role of P2X7R in glioblastoma tumor cell proliferation, the recruitment of microglia and the promotion of an inflammatory microenvironment^27–29^. Inhibition of this receptor has been demonstrated to decrease tumor cell number in human glioma cultures^16,30^. These results support the notion that P2X7R plays a trophic role in glioblastoma and highlights the potential of P2X7R as a therapeutic target to reduce the progression of glioblastoma.

GM-CSF, also known as colony-stimulating factor 2 (CSF2), is a glycoprotein secreted, for example by several innate and adaptive immune cells, including macrophages^31^. It is produced in response to pro-inflammatory cytokines, and functions as a cytokine promoting proliferation, migration and development of several myeloid populations including neutrophils, eosinophils and macrophages through well characterized inflammatory pathways such as Janus Kinase/Signal Transducer and Activator of Transcription (JAK/STAT) signaling and NFκB activation^32–34^. In the cancer setting, GM-CSF has been described to have dual roles, showing both pro-tumor and anti-tumor effects^35^. It is currently a key cytokine being investigated in the emerging field of cancer immunotherapy, due to its broad range of immunostimulatory effects acting on several different immune cells^36^. Recent cancer vaccines utilize this by inducing GM-CSF secretion from inactivated tumor cells to stimulate dendritic cells and macrophages to increase antigen presentation and the immune system’s ability to recognize and destroy tumor cells^37,38^. However, GM-CSF has also been shown to promote tumor progression, possibly via activation of Signal transducer and activator of transcription 3 (STAT3) or upregulation of vascular endothelial growth factor (VEGF)^35^, and decreasing GM-CSF levels has been shown to suppress glioblastoma cell growth indicating a potential stimulatory effect on glioblastoma progression^39^.

To investigate the underlying causes and pathological processes in glioblastoma, several experimental models have been utilized, the most common being cell lines. Whilst there are limitations in the use of cell lines such as genetic changes occurring upon culture and inherent differences to the in vivo conditions, cell lines are still useful as initial experimental models, where results can be subsequently replicated in animal models or human samples^40,41^. The human U251 glioblastoma cell line is one of the most thoroughly investigated glioblastoma cell lines and allows for specific pathways and mediators to be investigated in vitro^42^, with previous studies having already investigated aspects of P2X7R biology in this model^43^. Use of the U251 cell line allows for rapid and repeatable investigations and can provide valuable insight into tumor characteristics in culture^42^.

From the above, both P2X7R and GM-CSF have been implicated in tumor pathogenesis; however, a connection between them has not been investigated. It is also unclear if GM-CSF could have direct functional effects on tumor cells in the absence of immune cells. Therefore, the objective of this study was to investigate the potential link between P2X7R and GM-CSF and determine if antagonism of P2X7R and GM-CSF neutralization could serve as possible means of reducing tumor cell proliferation.

## Results

### P2X7R and GM-CSFRα are expressed in U251 cells

**Figure 1:**
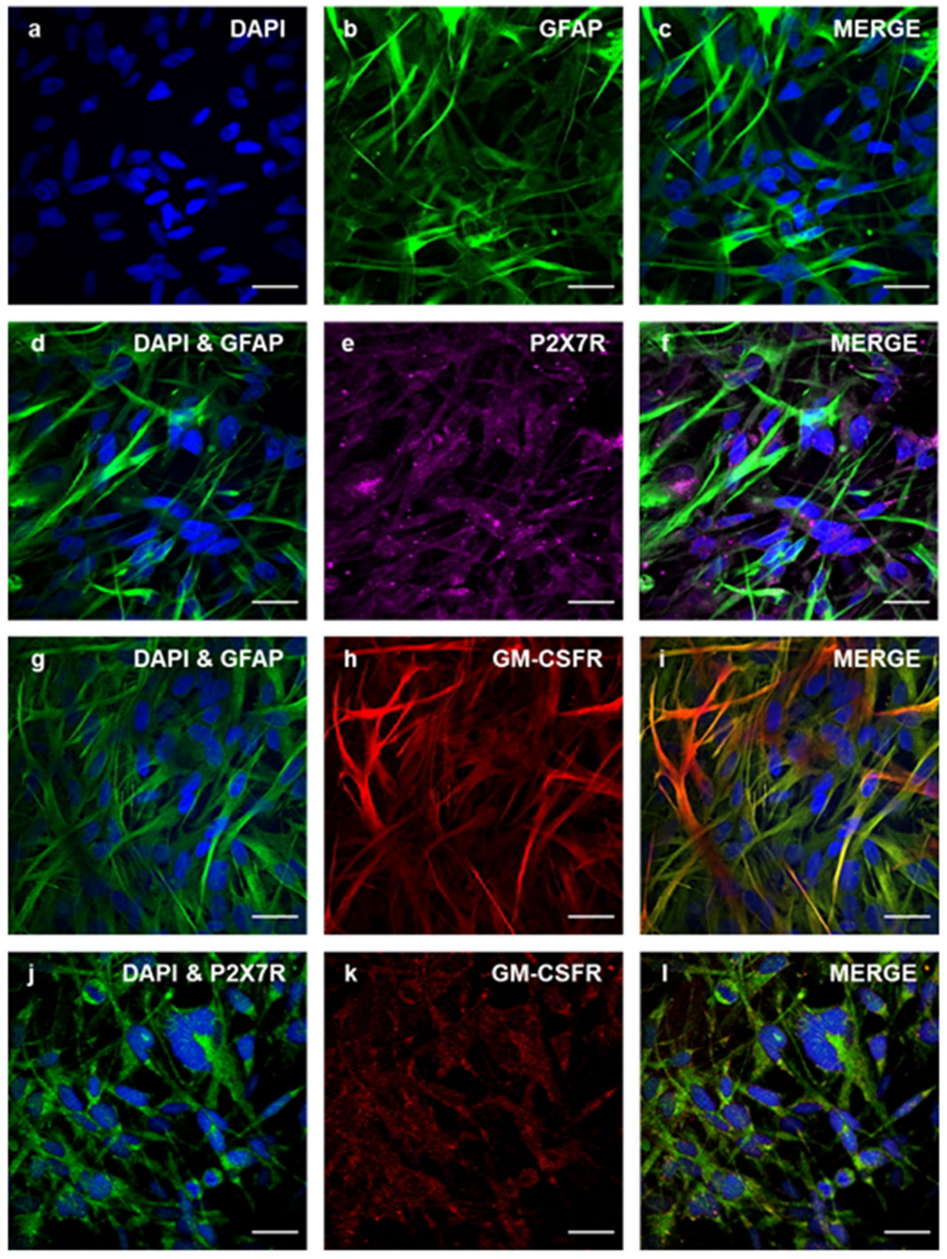
Expression of Glial fibrillary acidic protein (GFAP), P2X7R and GM-CSFRα in the U251 cell line. (**a**) DAPI nuclear stain (blue), (**b**) GFAP (green), (**c**) DAPI and GFAP overlayed expression, (**d**) GFAP and DAPI, (**e**) P2X7R (purple), (**f**) Co-localisation of GFAP and P2X7R expression. (**g**) DAPI and GFAP. (**h**) GM-CSFRα (red). (**i**) Co-localisation of GFAP and GM-CSFRα. (**j**) DAPI and P2X7R. (**k**) GM-CSFRα (red). (**l**) Co-localisation of P2X7R and GM-CSFRα expression. Scale bar = 33 μm for all images.

Immunocytochemistry was used to show expression of P2X7R and GM-CSFα in U251 cells (Fig. 1). P2X7R (Fig. 1e) and GM-CSFRα (Fig. 1h) were both expressed individually and co-expressed with GFAP, a marker of glioma cells (Fig. 1f,i). Furthermore, P2X7R (Fig. 1j) and GM-CSFRα (Fig. 1k) were shown to be co-localized in the U251 cells (Fig. 1l) confirming expression in U251 cells.

### The P2X7R ion channel is functional in U251 cells

**Figure 2:**
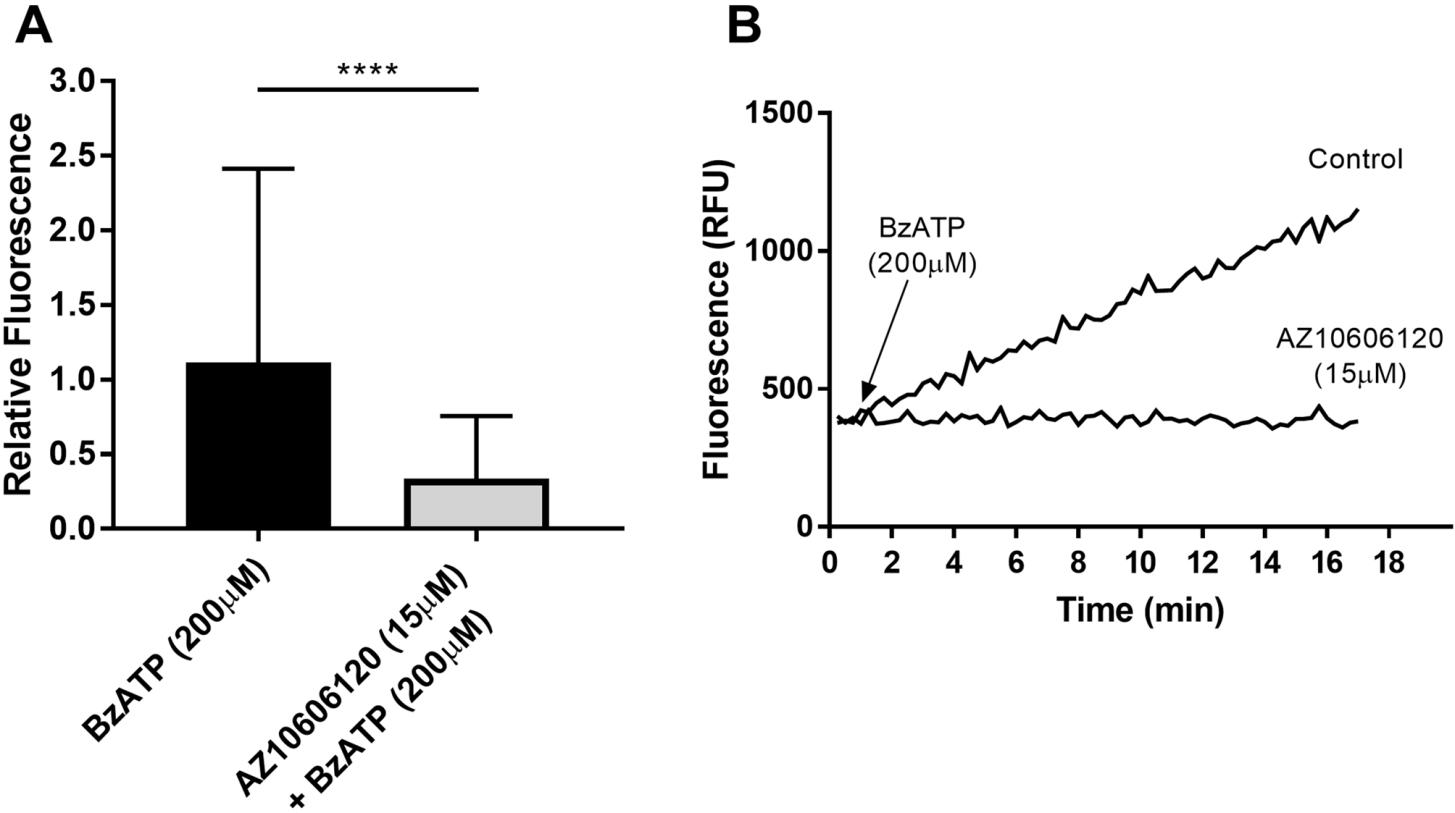
P2X7R is functional in the U251 cells. (**A**) In response to BzATP (200 µM) stimulation there was an increase in Fluo-4 AM fluorescence intensity indicative of P2X7R channel activity. This response was significantly reduced upon pre-treatment with P2X7R antagonist AZ10606120 (15 µM). A total of 10–22 cells (as regions of interest per field were selected) for fluorescence measurement changes, n = 8 untreated samples (190 cells), and n = 7 AZ106 treated samples (136 cells). Data is expressed as mean ± SEM. ****P < 0.0001. Mann Whitney U Test. (**B**) Characteristic race of fluorescence change over time after stimulation with BzATP (200 μm) and treatment with AZ10606120 (15 μM).

The synthetic calcium indicator Fluo-4 AM was used to measure P2X7R channel responses where intracellular rises in calcium are measured as a change in fluorescence intensity. P2X7R channel function was present in the U251 cells upon agonist stimulation (BzATP) (Fig. 2). Treatment with the P2X7R inhibitor, AZ10606120, significantly reduced this response (P < 0.0001).

### GM-CSF mRNA and protein expression are decreased by AZ10612010

**Figure 3:**
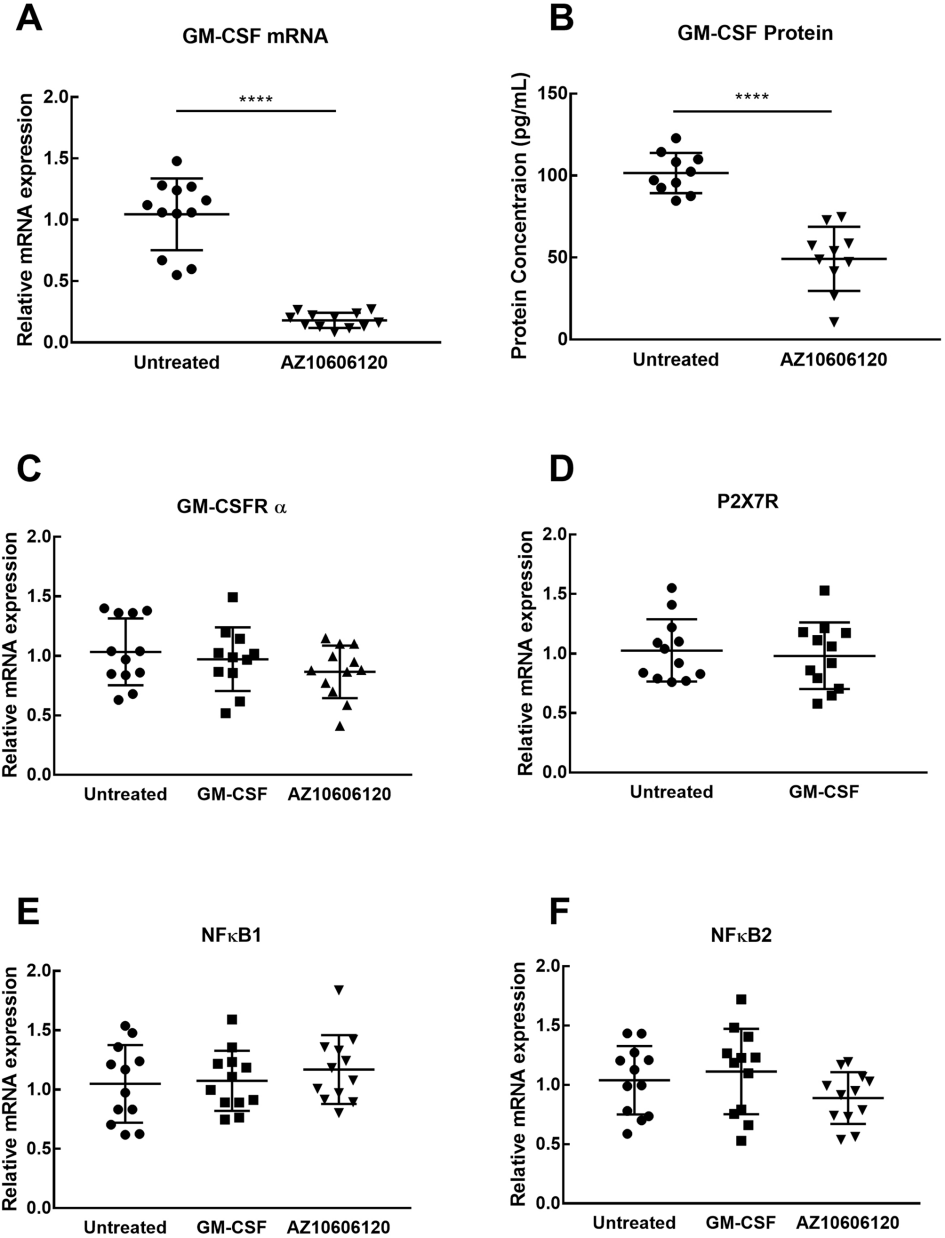
Alterations in mRNA and protein expression of key mediators and receptors of neuroinflammation under treatment with P2X7R antagonists in U251. (**A**) GM-CSF mRNA expression was significantly decreased after treatment of AZ10606120 compared to untreated control. (**B**) GM-CSF protein levels were also significantly decreased in samples treated with AZ10606120. There was no significant difference in GM-CSFRα (**C**) P2X7R (**D**), NFκB1 (**E**), and NFκB2 (**F**) mRNA expression across all treatments. Data is represented as mean ± SEM, n = 10–12 (**A**,**B**,**D**) Unpaired t-test, (**C**,**E**,**F**) 1-way ANOVA, ****P < 0.0001.

To support the purported link between GM-CSF and P2X7R, both mRNA and protein levels of GM-CSF were investigated upon treatment with AZ10606120. Treatment with AZ10606120 resulted in significant decreases in GM-CSF mRNA (P < 0.001) and protein expression (P < 0.001) compared to controls (Fig. 3A,B). mRNA expression was investigated for a number of key potential signaling components (P2X7R, GM-CSF, GM-CSFRα, GM-CSFRβ, NFκB1 and NFκB2) under different treatment conditions (Untreated, GM-CSF 15 ng/mL; AZ10606120 15 μM; n = 12) in U251 cells (Fig. 3C–F). There was no significant difference in expression between treatment conditions; GM-CSFRβ mRNA expression was undetectable by qPCR (Data not shown). There was also no significant difference in P2X7R (Fig. 3D), NFκB1 (Fig. 3E) and NFκB2 (Fig. 3F) mRNA expression between treatments.

### P2X7R antagonist significantly decreases cell proliferation

**Figure 4:**
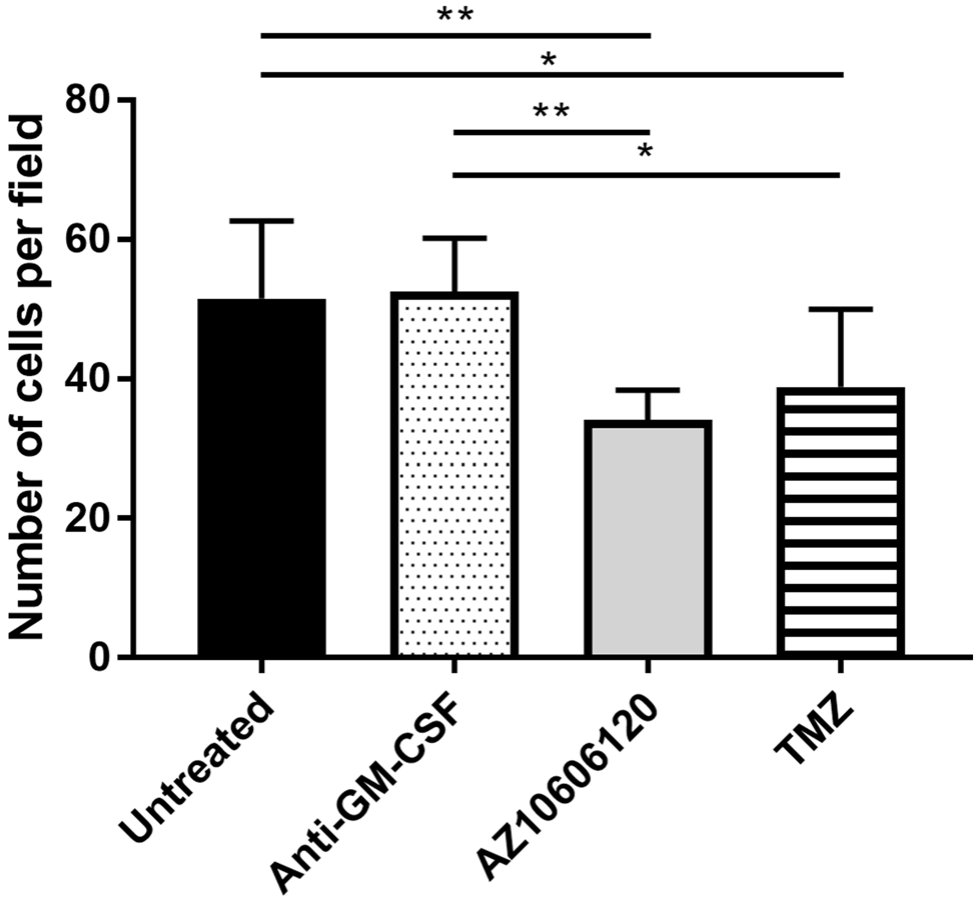
Cell proliferation is significantly decreased by P2X7R antagonism. A significant reduction in cell proliferation was seen after both AZ10606120 and TMZ treatment compared to untreated cells (n = 8, 25 fields/n). Data is represented as mean ± SEM. One-way ANOVA *P < 0.05; **P < 0.01.

Treatment with AZ10606120 and anti-GM-CSF mAb were compared to treatment with Temozolomide, the conventional chemotherapy agent, to determine the effects on cell proliferation. Treatment with anti-GM-CSF mAb had no significant effect on tumor proliferation compared to untreated cells. Conversely, treatment with AZ10606120 (P2X7R antagonist) significantly reduced cell proliferation compared to both untreated and anti-GM-CSF mAb treatment (P > 0.01). Treatment with Temozolomide also significantly reduced cell proliferation compared to untreated and anti-GM-CSF treatment mAb, though to a lesser extent than the reduction seen with AZ10606120 treatment (Fig. 4).

## Discussion

**Figure 5:**
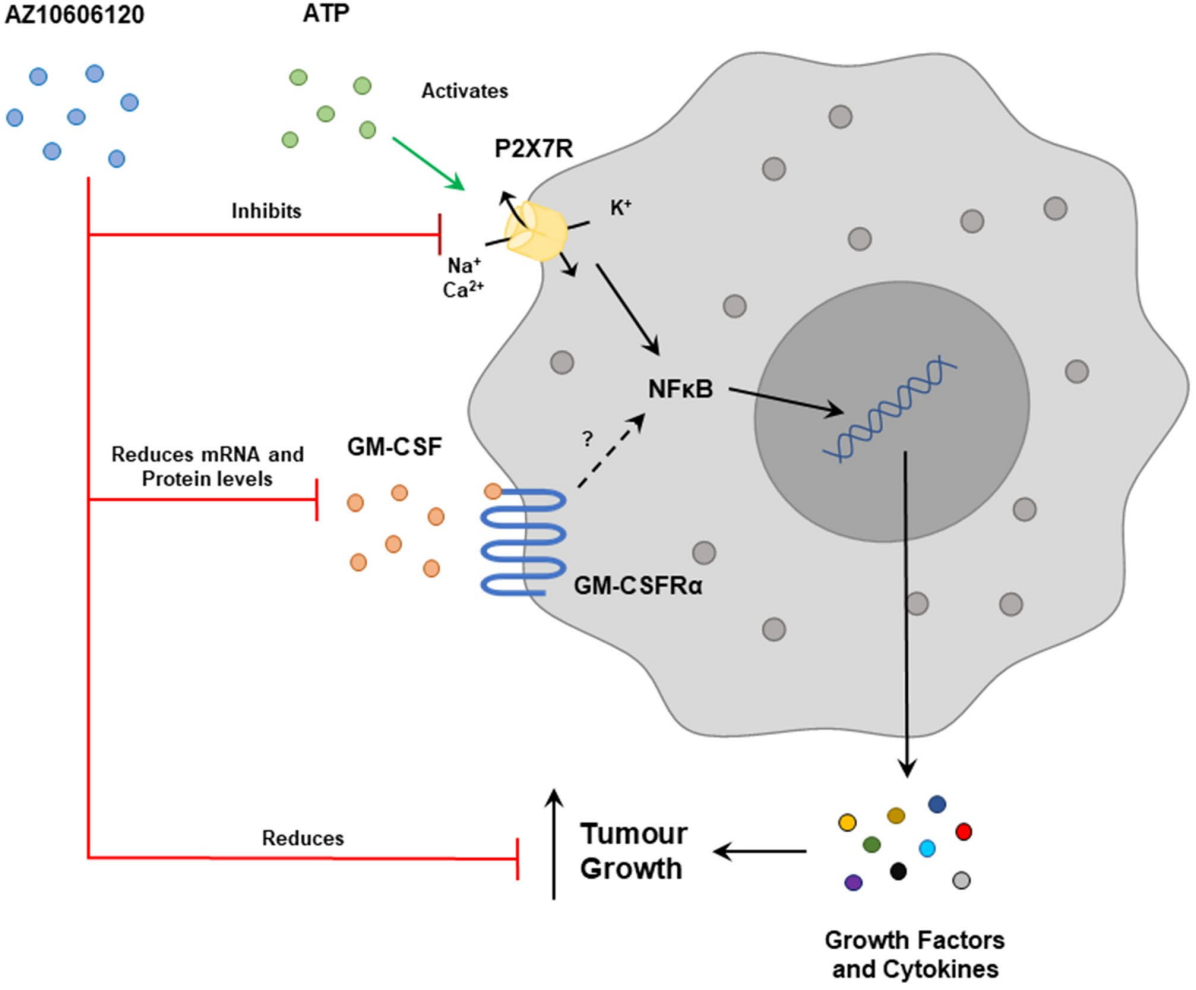
Diagrammatic representation of AZ10606120 interactions with P2X7R and GM-CSF and its effects on tumour growth. P2X7R acts through NFκB to release cytokines and growth factors that can increase tumour growth. AZ10606120 is an antagonist that inhibits P2X7R and leads to a reduction in GM-CSF production in the U251 cells as well as inhibiting tumour proliferation. GM-CSFRβ is not expressed in the U251 cell line, and a role of GM-CSFRα signalling has not yet been identified.

The results of this study identified expression of both P2X7R and GM-CSFRα in the U251 glioblastoma cell line and established that the P2X7R ion channel is functional in this setting. It highlights a previously undescribed mechanistic link between P2X7R and GM-CSF through changes in mRNA expression and protein levels. Lastly, it demonstrates the potential therapeutic benefit of the P2X7R antagonist AZ10606120 at reducing tumor cell proliferation (Fig. 5).

P2X7R and GM-CSFRα expression were demonstrated in U251 cells by immunocytochemistry and confocal microscopy. The antibodies used have been validated previously in multiple studies^24,44,45^. These findings are consistent with previous published data that have demonstrated that P2X7R is present in other glioblastoma cell lines, human glioma samples and orthotopic glioma animal models^16,27,29,46^. The GM-CSF receptor consists of both an α and a β subunit which are necessary for receptor function and signaling^33^. Our data demonstrates the presence of GM-CSFRα, but we could not detect GM-CSFRβ in U251 cells by qPCR. Previous studies using flow cytometry reported that GM-CSFR was not present in the U251 cell line, however the specific receptor subunit investigated was not stated^47^. A more recent study using semi-quantitative RT-PCR identified that GM-CSFRα is expressed in both human glioblastoma cultures and to a lesser extent in normal brain tissue, although the cell of origin was not stated^48^. Lack of GM-CSFRβ abolishes GM-CSF signaling^49^, as it has been shown, for example, that mice with inactivation of the β subunit gene have a similar phenotype to GM-CSFR^−/−^ knockout mice^50^. Therefore, the lack of changes seen from GM-CSF treatment could be attributed to lack of signaling. Further investigation of the role of expression and function of each subunit of the receptor in the context of glioblastoma is required.

The P2X7R ion channel was identified as functional, measured by monitoring intracellular Ca^2+^ dynamics upon P2X7R stimulation (BzATP). The channel responses observed in the U251 cell line were P2X7R specific as they were significantly limited by the specific antagonist of the receptor, AZ10606120^51^. To our knowledge, this is also the first study to demonstrate expression of GM-CSFRα and functional P2X7R in the U251 glioblastoma cell line.

GM-CSF gene and protein expression were significantly decreased upon treatment with AZ10606120. Whilst the exact relationship between P2X7R and GM-CSF is not described, these results confirm that P2X7R activity can influence expression of GM-CSF (Fig. 5). Conversely, P2X7R mRNA expression was not altered by treatment with GM-CSF, presumably due to the lack of the GM-CSFR β subunit in these cells. GM-CSF has been shown to be upregulated in glioblastoma and associated with tumor growth, and demonstrated to induce expression of the IL-4 receptor in glioma myeloid cells, leading to immunosuppression in the tumor microenvironment^52^. Reduced levels of GM-CSF have been associated with suppression of tumor growth in glioblastoma in an immune-independent manner^39^. Interestingly, GM-CSF administration has also shown anti-tumor effects, being able to regulate immune responses through recruitment and activation of dendritic cells to increase tumor antigen presentation leading to immune-mediated removal of cancer cells^35^. This immunostimulatory function of GM-CSF is also currently being investigated for use in cancer vaccines, with GM-CSF-transduced inactive glioma cells having been shown to increase survival in glioma mouse models, by stimulating immune mediated clearance of tumor antigens^53^. Thus the role of GM-CSF in the glioblastoma tumor setting is dependent on the context of the surrounding microenvironment, and its role in cancer pathology, or specifically in glioblastoma progression, is not fully understood.

Lastly, mRNA expression of NFκB subunits were not altered by treatment with GM-CSF or P2X7R antagonists. NFκB is activated upon P2X7R channel function (potassium efflux and calcium influx), resulting in degradation of the inhibitor of κB to release NFκB; therefore their unchanged mRNA expression via P2X7R inhibition is in line with this mechanism^54^. The GM-CSFR has also been reported to induce NFκB production; however the lack of GM-CSFR signalling could indicate why no changes were seen^55,56^ (Fig. 5). Lastly, NFκB function has also been shown to increase GM-CSF production by controlling its transcription^57^ highlighting both a potential positive feedback loop of GM-CSF production via NFκB, as well as a potential pathway linking P2X7R activation to release of NFκB and subsequent production of GM-CSF. Whilst the negative results do not rule out a role of NFκB in a mechanistic pathway involving P2X7R and GM-CSF^56–58^, they do indicate that inhibition of P2X7R and increasing GM-CSF do not result in altered NFκB mRNA expression Future research to further characterise this pathway in both glioblastoma as well as other non-cancerous settings is necessary to both understand this relationship and investigate if it could be utilised in other disease settings.

A neutralizing anti-GM-CSF mAb, previously demonstrated to neutralize GM-CSF induced cell proliferation^59^, was used to lower GM-CSF concentrations in the U251 cell culture, mimicking one downstream consequence of P2X7R inhibition. Reducing GM-CSF in this way caused no significant changes whilst treatment of cells with AZ10606120 significantly reduced cell proliferation. Therefore, whilst AZ10606120 reduces tumor cell proliferation and GM-CSF concentration, removing GM-CSF itself is not sufficient to cause this effect. Thus, it is likely that other mediators are responsible for this effect of P2X7R antagonism on cell proliferation. These results are consistent with previous research demonstrating that P2X7R is associated with a number of downstream pro-inflammatory pathways^15,20,22,25^ and hence effects on GM-CSF could be one of many such mechanisms. The effect of P2X7R antagonism in other cancer settings have been demonstrated in vivo where AZ10606120 treatment inhibited tumor growth mouse models of pancreatic cancer^19^ and mesothelioma^17^. Temozolomide, the conventional therapy used in glioblastoma, also resulted in decreased cell proliferation, but with less efficacy when compared to treatment with AZ10606120. Considering the toxic side effects of Temozolomide treatment, these results highlight the potential for AZ10606120 as a possible safer, more efficacious treatment for glioblastoma.

## Conclusion

This study describes, for the first time, a link between P2X7R and GM-CSF and the effect of P2X7R antagonism on cell proliferation in human glioblastoma cell line U251. In U251 glioblastoma cell line, P2X7R is expressed and functional, and inhibition of the receptor with AZ10606120 caused a decrease in GM-CSF mRNA and protein expression. Importantly, and compared to conventional chemotherapy Temozolomide, treatment of the U251 glioblastoma cell line with P2X7R antagonist AZ10606120 resulted in a significant decrease in glioma cell proliferation. This study highlights an important link between P2X7R and GM-CSF in glioblastoma and revealed a potentially exciting new therapy in AZ10606120.

## Methods

### U-251 MG cell culture

The U-251 MG human glioblastoma cell line (U251; Sigma) was maintained in Dulbecco’s modified Eagle’s Medium (DMEM; Lonza), supplemented with 10% heat-inactivated Fetal Bovine Serum (FBS; Life Technologies), penicillin–streptomycin (5,000 U/mL; Life Technologies), 1% non-essential amino acids (Sigma) and sodium pyruvate (1 mM; Sigma). Cells were cultured in a humidified incubator at 37 °C, 5% CO_2_/95% O_2_ and routinely passaged at 80% confluency.

### Reagents

Cells were grown to 70% confluency and then treated for 72 h unless specified otherwise with the following reagents: human GM-CSF (15 ng/mL; R&D Systems), AZ10606120 (15 μM; Sigma), anti-human GM-CSF monoclonal antibody (20 ng/mL; R&D Systems) and Temozolomide (TMZ; 50 μM; Sigma).

### Immunocytochemistry

Cultured cells were fixed with 50% acetone/methanol at − 20 °C for 15 min, washed and immersed in 2% Bovine Serum Albumin (BSA; Sigma) for 1 h at 37 °C. Samples were incubated with the following primary antibodies for 48 h at 4 °C: anti-GFAP (Glial fibrillary acidic protein; a commonly used marker to identify human glioma cells^60^) Alexa Fluor 488 conjugate (1:200; Thermo Fisher), anti-GM-CSFRα Alexa 594 conjugate (1:200; R&D Systems), mouse anti-human GM-CSFRα non conjugate (1:100; R&D Systems), anti-P2X7R FITC (Fluorescein isothiocyanate) conjugate (1:100; Sigma), goat anti-P2X7R non-conjugated (1:100; Quantum Scientific) and rabbit anti-GFAP non conjugated (1:400; Dako). The following secondary antibodies were used: Alexa Fluor 488 (1:200; Invitrogen), Texas Red 633 anti-goat (1:200; Invitrogen) and Texas Red 594 anti-rabbit (1:200; Invitrogen). Samples were washed and counterstained with 5 μM of 4′,6-diamidino-2-phenylindole (DAPI) nuclear stain (Invitrogen), then mounted and imaged using a Nikon A1r confocal microscope. All experiments were conducted with negative controls (in the absence of primary antibody; isotype control).

### Live cell imaging

Cells were grown on 18 mm^2^ glass coverslips at a density of 1 × 10^6^. The coverslips of cells were placed in 4-(2-hydroxyethyl)-1-piperazineethanesulfonic acid (HEPES) buffer and incubated with 5 μM of Fluo-4 AM (Molecular Probes, Life Technologies) for 1 h at room temperature. The cells were then imaged using a Ziess LSM 510 M META fluorescence confocal microscope. To elicit P2X7R channel activity the cells were stimulated with P2X7R agonist BzATP (200 µM; Sigma) and the change in fluorescence intensity was measured using the TimeSeries component of the software where images were captured every 15 s. To make sure that the changes in fluorescence were P2X7R specific some coverslips were preincubated with P2X7R antagonist AZ10606120 (15 µM) for 15 min prior to the above. Channel function was quantified as change in fluorescence before and after addition of BzATP (ΔF) and expressed as the relative change in fluorescence intensity (ΔF/F_min_) which correlates with changes in Ca^2+^ concentration as a function of ion movement through the P2X7R ion channel.

### RNA extraction, cDNA synthesis and quantification

Cultured cells were treated with human GM-CSF (15 ng/mL), AZ10606120 (15 μM) or left as untreated controls. At termination cells were gently scraped, centrifuged and frozen at − 80 °C. Total RNA was isolated using a QIAGEN RNeasy Plus Mini Kit (QIAGEN) and a QIAcube robotic workstation (QIAGEN) following the manufacturer’s protocol. 2 μg of total RNA per sample were converted to cDNA using the QuantiTect Reverse Transcription Kit (QIAGEN) containing Quantiscript Reverse Transcriptase, Quantiscript RT Buffer and RT primer Mix following the manufacturer’s protocol. 50 ng cDNA per sample was analyzed using catalogued human Taqman gene expression assays (Life Technologies) for P2X7R (assay ID Hs00175721_m1), GM-CSF (CSF2; assay ID Hs00929873_m1), NF-kappa B Nuclear Factor subunit 1 (NFκB1; assay ID Hs00765730_m1), NF-kappa B Nuclear Factor subunit 2 (NFκB2; assay ID Hs01028890_g1), GM-CSF receptor subunit α (GM-CSFRα; assay ID Hs00531296_g1) GM-CSF receptor subunit β (GM-CSFRβ; assay ID Hs00166144_m1) and 5 housekeeping genes, namely Glyceraldehyde 3-phosphate dehydrogenase (GAPDH; assay ID Hs02758991_g1), β-actin (assay ID Hs01060665_g1), Succinate dehydrogenase complex, subunit A (SDHA; assay ID Hs00188166_m1), Peptidylprolyl isomerase A (PPIA; assay ID Hs99999904_m1) and 60S ribosomal protein L13a (RPL13A; assay ID Hs03043885_g1). qPCR was carried out using a QuantStudio7 Flex Real-Time PCR system (Thermo-Fisher). Relative assessment of mRNA was performed using the ΔΔC_T_ method with relative expression compared to housekeeping genes^61^.

### Enzyme linked immunosorbent assay (ELISA)

Cultured cells were treated with AZ10606120 (15 µM) or left as untreated controls for 72 h, and supernatant collected. ELISA was performed using the Human GM-CSF Quantikine ELISA Kit (R&D Systems) with 200 μL per sample (in duplicate) loaded into the ELISA plate following manufacturer’s protocol. Optical density was measured using a Multiskan Spectrum microplate reader (Thermo-Fisher Scientific) set to 450 nm (with correction at 540 nm) with SkanIt Software 2.4.2. GM-CSF protein levels were determined from a standard curve constructed from a dilution series of standard absorbance values.

### Cell counts

Samples treated with AZ10606120 (15 μM), anti-human GM-CSF (20 ng/mL), TMZ (50 μM) or control (untreated) were fixed on 18 mm coverslips with 50% acetone/methanol for 15 min at − 20 °C, washed with Phosphate Buffered Saline (PBS; Sigma), incubated with 5 μM of DAPI nuclear stain (dark, 1 h, room temperature) and then washed and mounted. Images were taken using an Olympus IX-81 fluorescence microscope with a 40× air objective lens. Twenty-five random fields were imaged per sample, and cells counted using Metamorph Imaging Software (Universal Imaging Corporation).

### Statistical analysis

All statistical tests were completed using GraphPad Prism 7 software. D'Agostino & Pearson normality test was used to determine normality. Non-parametric data was analyzed by Mann Whitney U test and Kruskal–Wallis test with post hoc Dunn’s multiple comparisons depending on the number of comparisons. Unpaired t-tests and one-way Analysis of Variance (ANOVA) with post hoc Tukey’s HSD tests were utilized for parametric data. Data was expressed as mean ± S.E.M with statistical significance set at P < 0.05.

## Data Availability

The data that support the findings of this study are available from the corresponding author, Dr. Mastura Monif, upon reasonable request.

## Abbreviations

ANOVA: Analysis of variance
ATP: Adenosine triphosphate
BzATP: 3′-*O*-(4-Benzoyl)benzoyl ATP
CNS: Central nervous system
CSF2: Colony-stimulating factor 2
DAPI: 4′,6-Diamidino-2-phenylindole
DMEM: Dulbecco’s modified eagles medium
ELISA: Enzyme linked immunosorbent assay
FITC: Fluorescein isothiocyanate
GAPDH: Glyceraldehyde 3-phosphate dehydrogenase
GFAP: Glial fibrillary acidic protein
GM-CSF: Granulocyte-macrophage colony-stimulating factor
GM-CSFR: Granulocyte-macrophage colony-stimulating factor receptor
GM-CSFRα: GM-CSF receptor subunit α
GM-CSFRβ: GM-CSF receptor subunit β
HEPES: 4-(2-Hydroxyethyl)-1-piperazineethanesulfonic acid
IL-1β: Interleukin 1 beta
JAK/STAT: Janus kinase/signal transducer and activator of transcription
NF-κB: Nuclear factor kappa-light-chain-enhancer of activated B cells
NFκB1: NF-kappa B nuclear factor subunit 1
NFκB2: NF-kappa B nuclear factor subunit 2
P2X7R: Purinergic P2X Receptor 7
PBS: Phosphate buffered saline
PPIA: Peptidylprolyl isomerase A
qPCR: Quantitative polymerase chain reaction
RPL13A: 60S ribosomal protein L13a
SDHA: Succinate dehydrogenase complex, subunit A
STAT3: Signal transducer and activator of transcription 3
TMZ: Temozolomide
VEGF: Vascular endothelial growth factor
